# N-Butanol Fraction of Wenxia Formula Extract Inhibits the Growth and Invasion of Non-Small Cell Lung Cancer by Down-Regulating Sp1-Mediated MMP2 Expression

**DOI:** 10.3389/fphar.2020.594744

**Published:** 2020-11-30

**Authors:** QianYu Bi, MengRan Wang, Fang Zhao, Meng Wang, XiangJun Yin, JiaZhao Ruan, DeLong Wang, XuMing Ji

**Affiliations:** ^1^ School of Basic Medical Science, Zhejiang Chinese Medical University, Hangzhou, China; ^2^ College of Chinese Traditional Medicine, Shandong University of Traditional Chinese Medicine, Jinan, China; ^3^ Department of Medicine, Jining NO.1 People's Hospital, Jining, China; ^4^ Academy of Chinese Medical Science, Zhejiang Chinese Medical University, Hangzhou, China; ^5^ Key Laboratory of Neuropharmacology and Translational Medicine of Zhejiang Province, Hangzhou, China

**Keywords:** N-butanol fraction of wenxia formula extract, wenxia formula, non-small cell lung cancer, Sp1, MMP2, MMP9

## Abstract

Lung cancer is the most commonly diagnosed cancer and the leading cause of cancer death. It is necessary to develop effective anti-lung cancer therapeutics. Wenxia Formula (WXF), an empirical traditional Chinese herbal formula, has been reported to have significant antitumor activity. In this study, to further clarify the material basis of the anti-tumor effect of WXF, we investigated the cytotoxic effect of the N-butanol fraction of Wenxia Formula extract (NWXF) against two lung cancer and one normal human cell lines. The chemical profile of NWXF was characterized by UPLC/Q-TOF-MS analysis and a total of 201 compounds with mzCloud Best Match of greater than 70 were identified by using the online database mzCloud. To address the functional role of NWXF, we assessed cell proliferation, migration and invasion capabilities. Subcutaneous xenografts were constructed to determine the effect of NWXF in vivo. The results showed that NWXF effectively inhibited the proliferation and migration of non-small cell lung cancer (NSCLC) cells with little toxic effects on human bronchial epithelial cells. Meanwhile, orally administered NWXF exhibited prominent dose-dependent anti-tumor efficacy in vivo. Mechanistically, NWXF significantly downregulated MMP9 and Sp1-mediated MMP2 expression. In conclusion, NWXF might be a promising candidate for treatment of human lung cancer.

## Introduction

Non-small cell lung cancer (NSCLC) is the predominant form of lung cancer, accounting for about 85% of primary lung cancer cases (Chen et al., 2016). It has been the most common cancer and the leading cause of cancer-related death globally, and its incidence is still increasing (Bray et al., 2018). Tumor metastasis is the leading cause of mortality in lung cancer patients and therapeutic approaches against metastasis are almost ineffectual. The essential features of the metastatic process include tumor cell invasion and migration. The capacity of cancer cells to escape from the primary tumor, move into lymphatic and blood vessels and then invade other tissues is advanced by matrix metalloproteinases (MMPs) of which one of the functions is degrading the extracellular matrix (ECM) (Gonzalez-Avila et al., 2020). MMPs disruption of ECM has significant impact on pathophysiological functions in condition of cancer. Moreover, numerous studies have demonstrated the ability of MMPs to promote all aspects of cancer hallmarks from cell proliferation to metastasis (Li K. et al., 2020; Laronha and Caldeira, 2020). Therefore, development of novel generation of anti-MMPs candidates is a promising and urgent task for NSCLC treatment (Laronha et al., 2020).

Wenxia Formula (WXF), an empirical herbal formula of traditional Chinese medicine for treating cancer, is composed of *Aconitum carmichaeli Debeaux, Rheum palmatum L.*, *Panax ginseng C.A.Mey* and *Angelica sinensis (Oliv.) Diels*. Previous studies have demonstrated that WXF is a potent anti-carcinogenic agent that inhibits multiple signaling pathways, inducing apoptosis and cell cycle arrest (Ji et al., 2016; Zhang et al., 2019b). To further clarify the material basis of the anti-tumor effect, WXF was extracted with 75% ethanol and then partitioned into four fractions: petroleum ether fraction, methylene chloride fraction, ethyl acetate fraction and n-butanol fraction. Among them, we found that N-butanol fraction of Wenxia Formula extract (NWXF) had good anti-tumor effect *in vitro*. In the present study, we evaluated the molecular mechanism by which NWXF inhibited NSCLC cells migration. This dissertation provided a scientific basis so as to further clarify the material basis of the anti-tumor effect of NWXF. It also enriched the content of WXF active ingredients’ research.

## Materials and Methods

### Cell Lines and Cell Culture

A549, H1299, and H460 cell lines were purchased from the Cell Bank of Type Culture Collection of Chinese Academy of Sciences (Shanghai, China). Human bronchial epithelial cells (HBE) were purchased from Procell Biotechnology (Wuhan, China). Cells were maintained in RPMI 1640 (Gibco, United States) supplemented with 10% FBS (Gibco, United States) and antibiotics (penicillin, 100 U/ml; streptomycin 100 μg/ml). All cells were cultured at 37°C in a humidified atmosphere of 5% CO_2_.

### Herbal Preparation

WXF consists of four kinds of Chinese medicinal herbs, including *Aconitum carmichaeli Debeaux* (3,000 g, Batch No. 20180609)*, Rheum palmatum L.* (3,000 g, Batch No. 20180609)*, Panax ginseng C.A.Mey* (2250 g, Batch No. 20180609), and *Angelica sinensis (Oliv.) Diels* (1,500 g, Batch No. 20180609)*.* All herbal materials were identified by Prof. F. Li. of Shandong University of Traditional Chinese Medicine. Detailed information on the drug materials and the scan of the vouchers were given in Supplementary Table S1. The WXF was smashed and successively extracted twice with 75% ethanol by ultrasonic method. The extracts were filtered, followed by concentration by rotary evaporation with reduced pressure at 60°C. Then, the extracts were concentrated for extraction with petroleum ether, methylene chloride, ethyl acetate, and n-butanol sequentially to obtain NWXF (about 98 g) for further tests.

For *in vitro* experiments, compounds were dissolved in DMSO (Solarbio, China) and stock solutions were stored at 4°C until being used. For *in vivo* experiments, NWXF was formulated in 0.5% methylcellulose (Solarbio, China).

### MTT Assay

The cytotoxic effects of NWXF on A549, H1299, H460, and HBE cells were determined by MTT assay. Cells (5 × 10^3^ cells/well) were incubated on 96-well plates in the presence or absence of NWXF (0, 125, 250, 500, 1,000 μg/ml) for 24 or 48 h. Then 20 μl of MTT (5 mg/ml) was added to each well and co-incubation for 4 h. After removing the MTT-containing medium, 150 μl of DMSO was added to solubilize the formazan. The optical density was measured at 490 nm by a microplate spectrophotometer (SpectraMax M3, Molecular Devices, United States)

### Cell Invasion and Migration Assay

A total of 1 × 10^5^ A549 cells were plated in the top chamber of transwell chamber (24-well insert; pore size 8 μm; Corning Costar, NY, United States). Each well was coated freshly with Matrigel (BD Bioscience, NJ, United States) before the invasion assay. Next, 600 μl 1,640 containing 10% FBS were added to the lower chamber. After incubation for 24 h, cells that had not migrated through the pores were removed with a cotton swab. Cells that had migrated through the membrane and stuck to the lower membrane surface were fixed with methanol and stained with 0.5% crystal violet. For the detection of migration, 1 × 10^5^ cells were added to the upper chamber without Matrigel coating, and 500 μl 1,640 containing 10% FBS in the lower chamber was used to induce the migration of the cells. After incubation for 24 h, the cells on the lower membrane were fixed with methanol and stained with 0.5% crystal violet. The number of cells that had migrated through the membrane were counted under a microscope.

### Western Blot Analysis

The expression levels of MMP2, MMP9, and Sp1 in lung cancer cells were determined by western blot analysis. Protein quantification was determined by the BCA protein assay. Then samples were equally loaded on 10% polyacrylamide gels electrophoresed at 120 V, electrotransferred to PVDF membranes, and probed with antibodies to MMP2 (1:1,000; lot number: Ab37150, Abcam, United States), MMP9 (1:1,000; lot number: GB12132–1, Servicebio, CHINA), Sp1(1:1,000; lot number: 227383, Abcam, United States), BAD (1:1,000; lot number: Ab32445, Abcam, United States). Finally, blots were quantified by densitometry using alphaease FC software.

### Chromatin Immunoprecipitation Assay

Cells were grown to 70% confluence in 10 cm culture dishes. A549 and H460 cells (6 × 106/well) were treated with NWXF for 24 h. Cells were cross-linked with 4% formaldehyde for 10 min, 125 mM glycine was added for 5 min at room temperature, and then the cells were washed and harvested in ice-cold PBS. The sonified chromatin was treated as described previously (Lin et al., 2017), using 2 μg of anti-Sp1. Purified DNA was used for PCR analysis. The human MMP2 and MMP9 promoter specific primer pairs used for amplification were as follows: MMP2, 5ʹ-ACA TCA AGG GCA TTC AGG AGC-3ʹ (forward) and 5ʹ-ACA GTC CGC CAA ATG AAC CG-3ʹ (reverse). MMP9, 5ʹ-GCA CGA CGT CTT CCA GTA CC-3ʹ (forward) and 5ʹ-GGT TCA ACT CAC TCC GGG AA-3ʹ (reverse). Chromatin immunoprecipitates from the proteins were amplified with PCR, normalized to input, and calculated as percentages of inputs.

### *In Vivo* Xenograft Studies

BALB/c female nude mice (4–6 weeks old, weighing 16–20 g) were maintained under specific pathogen-free conditions with constant temperature (23 ± 2°C) and controlled light (12 h light:12 h dark). The study was approved by the Institutional Animal Care and Use Committee (animal authorization reference number: SDUTCM2018111601) at Shandong University of Traditional Chinese Medicine (Shandong, China). Animal experimental procedures were strictly carried out in accordance with the Guide for the Care and Use of Laboratory Animals. All efforts were made to minimize animals’ suffering and to reduce the number of animals used. Xenografts were established via the subcutaneous injection of 2 × 10^6^ A549 (*n* = 25) into the right armpit of mice. Then, mice injected with A549-Luc were randomly divided into five groups (five mice/group) as follows: 1) the solvent control group; 2) the NXWF-low group that received 100 mg NWXF/kg/day by gavage, as determined by a previous study (data not shown); 3) the NWXF-medium group that received 200 mg NWXF/kg/day by gavage; 4) the NWXF-high group that received 400 mg NWXF/kg/day by gavage; and 5) the DDP (Cisplatin) group that received 5 mg DDP/kg/3 days by i.p. injection. Tumor volume was measured with digital calipers and small animal imaging with a modified ellipsoidal formula for volume (volume = 1/2 (length × width^2^). At 6 weeks after the subcutaneous injection, xenograft tumors were removed.

### Immunohistochemical Staining

Previously prepared paraffin-fixed nude mice tissue sections (3 mm) (normal and tumor) were processed for peroxidase (DAB) immunohistochemistry. After deparaffinization and rehydration using xylene and a series of weakening concentration of ethanol (95, 80, 70%), 50 ml of 1:1,000 dilution MMP2 and MMP9 primary antibody was added to each sample. The samples were stored overnight at 4°C. After being washed with water for 5 min, addition of peroxidase labeled polymer and substrate allowed the brown staining of the target proteins to be observed. The samples were counterstained by hematoxylin for 30 s.

### Analysis of Main Components of N-Butanol Fraction of Wenxia Formula Extract by Ultra-performance Liquid Chromatography Quadrupole Time-of-Flight Mass Spectrometry

UPLC/MS analysis was performed on Thermo Scientific™ Q Exactive high resolution mass spectrometer (Thermo Fisher Scientific, San Diego, CA) coupled with Thermo Fisher™ UltiMate 3000 RS system in negative ion mode. The sample solution was injected into a Thermo Hypersil GOLD column (100 × 2.1 mm, 1.9 μm) for UPLC separation. The mobile phase consisted of MeCN containing 0.1% (v/v) formic acid (A) and water containing 0.1% (v/v) formic acid (B). Linear gradient elution was applied (0–5 min, 2–20%A; 5–10 min, 20–50%A; 10–25 min, 50–95%A; 26–30 min, 2%A) at a flow rate of 0.3 ml/min. The column temperature was 35°C. For MS detection, accurate mass was maintained in full scan/data-dependent MS2 (full MS/dd-MS2) mode. The operating parameters were as follows: spary voltage, 3.8 kV; sheath gas pressure, 40 arb; Aux gas pressure, 10 arb capillary temperature, 300°C. MS data were acquired by CD 2.1 software (Thermo Fisher), and contrasted by using the online database mzCloud.

### Statistical Analysis

Data were expressed as the mean with the standard error, and the significance of the difference between two groups was determined using the two-tailed *t*-test. Multiple groups were analyzed with SPSS 26.0 by one-way ANOVA with Bonferroni posttests. For all statistical tests, *p* value < 0.05 were considered statistically significant.

## Results

### N-Butanol Fraction of Wenxia Formula Extract Could Inhibit the Proliferation of Human Lung Cancer Cell Line A549 and H460 Cells

**FIGURE 1:**
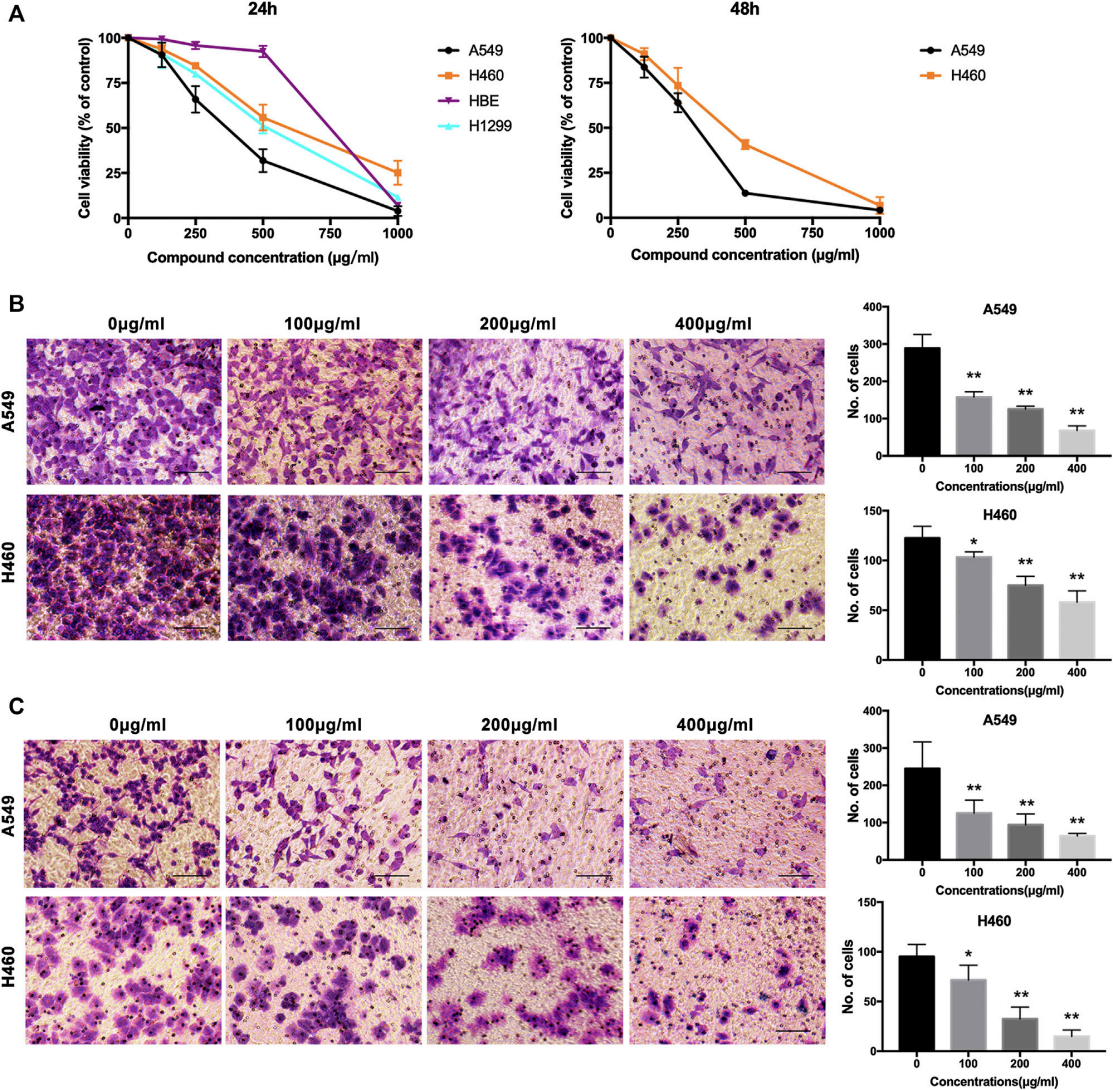
NWXF reduces viability and dampens migration and invasion in NSCLC cells. **(A)** Effects of NWXF on cell viability in A549, H1299, H460, and HBE cells. Cells were treated with various concentrations of NWXF for 24 and 48 h, respectively. Cell viability was assessed by MTT assays. **(B)** Migration assays and **(C)** invasion assays were performed in A549 and H460 cells after pre-treatment with indicated concentrations of NWXF for 24 h. Representative images are shown and the quantification of four randomly selected fields was shown below. Scale bar, 50 μm. Data were shown as mean ± SD. ^∗^ *p* < 0.05, ^∗∗^ *p* < 0.01 vs. control.

We evaluated the cytotoxic activity of NWXF against three cell types. Two cancer and one normal human cell lines (HBE, human bronchial epithelial cells) were used in this study. HBE cells were chosen as *in vitro* normal cell models. Cytotoxicity was measured by incubating the cells with serial dilutions of NWXF followed by MTT assay after 24 and 48 h. For every condition, cell viabilities were assessed in at least three independent experiments for every cell culture. As shown in Figure 1A, NWXF reduced viabilities of A549 and H460 cells in dose- and time-dependent manners. Moreover, the cytotoxicity of NWXF to HBE was lower than that of A549 cells. A549 and H460 cells were chosen for subsequent investigations.

### N-Butanol Fraction of Wenxia Formula Extract Could Inhibit the Migration and Invasion of Human Cell Line A549 Cells and H460 Cells

To explore the effects of NWXF on migration and invasion abilities of A549 and H460 cells, transwell migration and invasion assays were performed, respectively. The number of cells transferred to the lower membrane of the chamber in NWXF-treated groups was fewer than that in vehicle-treated groups in A549 (Figure 1B) and H460 (Figure 1C) cells.

### N-Butanol Fraction of Wenxia Formula Extract Inhibited Expression of Sp1, MMP2, and MMP9 in Human A549 and H460 Cells and Sp1 Was Responsible for N-Butanol Fraction of Wenxia Formula Extract Upregulation of MMP2 Transcription

**FIGURE 2:**
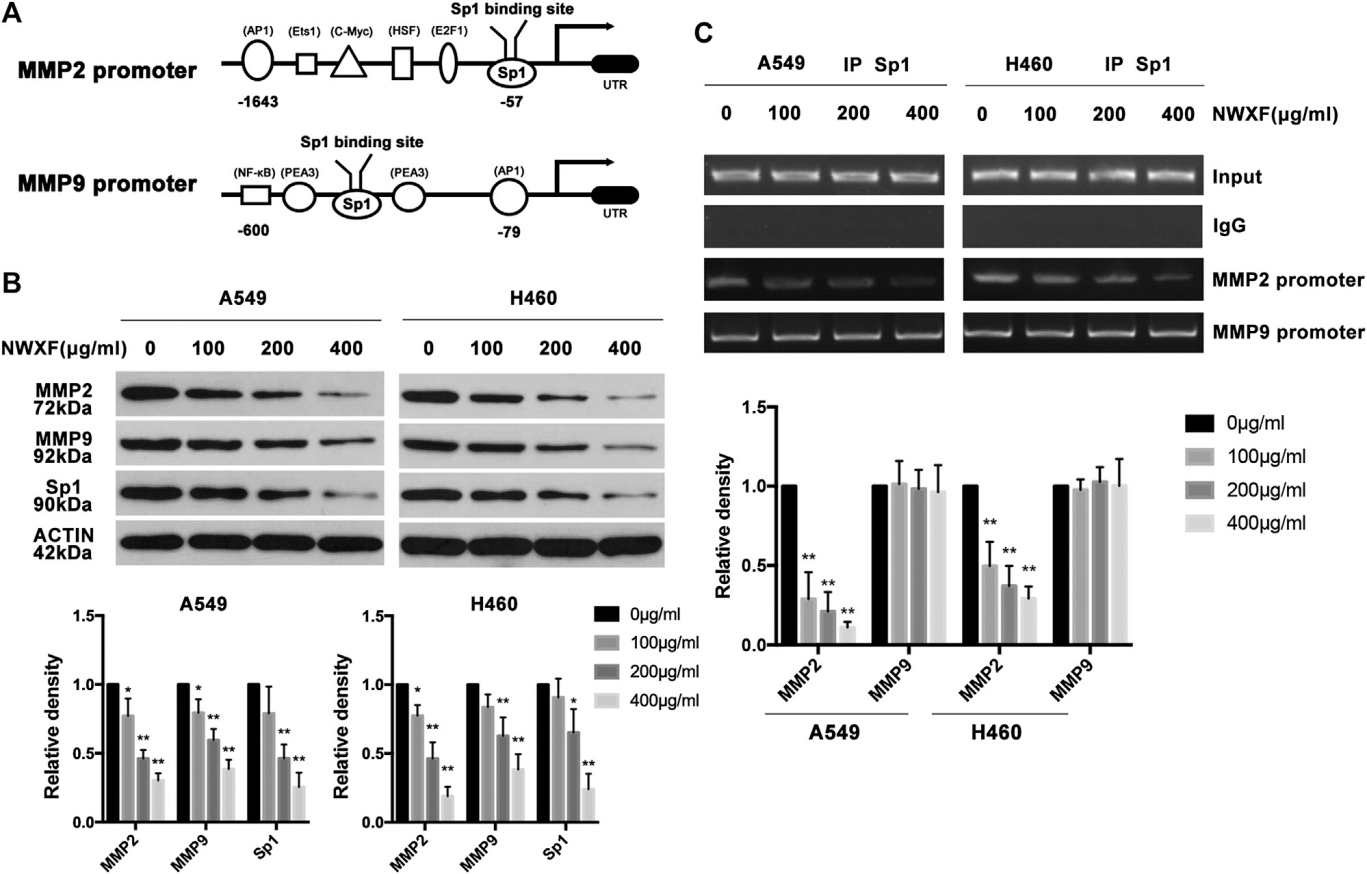
NWXF inhibits expression of Sp1, MMP2, and MMP9 in NSCLC cells. **(A)** Schematic representation of the putative Sp1 binding site on the promoter of MMP2 and MMP9. **(B)** A549 and H460 cells were treated with indicated concentrations of NWXF for 24 h, and then protein levels of Sp1, MMP2, and MMP9 were determined by immunoblotting. The relative protein levels were analyzed by alphaease FC software. **(C)** Chromatin immunoprecipitation (ChIP) analysis of the Sp1 binding to the MMP2 and MMP9 promoter region treated with NWXF for 24 h in A549 and H460 cells respectively. Data were shown as mean ± SD of three independent experiments. ^∗^ *p* < 0.05, ^∗∗^ *p* < 0.01 vs. control.

To determine the effects of NWXF on the expression of Sp1, MMP2 and MMP9 in human A549 and H460 cells, immunoblotting assay was performed. As shown in Figure 2B, NWXF dose-dependently lowered protein levels of total Sp1, MMP2, and MMP9 in both A549 and H460 cells. ChIP assay was performed to explore whether NWXF inhibited the binding activity of Sp1. We found that NWXF decreases the binding activity of Sp1 to reduce the transcription levels of MMP2 in A549 and H460 cells (Figure 2C).

### N-Butanol Fraction of Wenxia Formula Extract Inhibited Tumor Growth *In Vivo*

**FIGURE 3:**
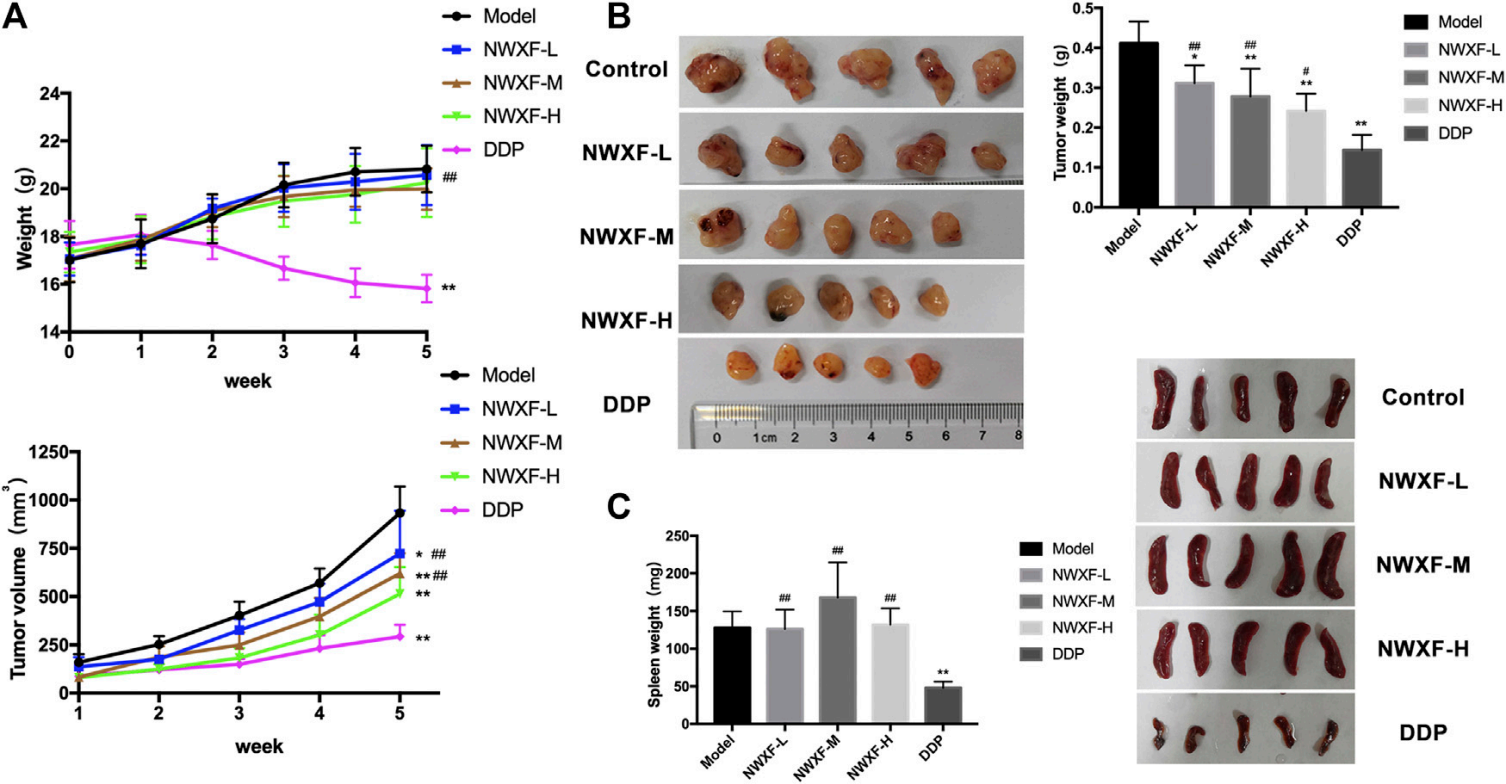
Anti-tumor activity of NWXF in subcutaneous xenografts model. **(A)** Mean body weights and tumor volume for indicated weeks were presented as mean ± SD (*n* = 5). **(B)** The pictures of tumors and the tumor weight. **(C)** The pictures of spleens and the spleen weight. ^∗^ *p* < 0.05, ^∗∗^ *p* < 0.01 vs. the control group. ^#^ *p* < 0.05, ^##^ *p* < 0.01 vs. the DDP group.

To further evaluate the tumor-suppressing effect of NWXF *in vivo*, a model for tumorigenicity of A549 NSCLC cells in nude mice was established. The effect of NWXF (200 mg/kg, 400 mg/kg, and 800 mg/kg) on tumor xenograft was examined (Figure 3). As anticipated, NWXF treatment significantly reduced the tumor sizes compared to the control. Specifically, the average tumor weight of control group was 0.41 ± 0.05 g, whereas the tumor weight in NWXF high/medium/low group was 0.24 ± 0.04 g, 0.28 ± 0.07 g, 0.31 ± 0.04 g, respectively. For the average tumor size, the control group showed 932.9 ± 137.8 mm^3^, whereas NWXF high/medium/low group was 514.2 ± 138.4 mm^3^, 618.8 ± 106 mm^3^, 722.2 ± 221.7 mm^3^, respectively (Figure 3A). Notably, NWXF treatment at the given concentration had little effect on the body weight and spleen weight of the mice (Figures 3B,C). Therefore, in animal model, NWXF also exhibited strong tumor suppression efficacy and low toxicity as it did *in vitro* cell culture system.

### N-Butanol Fraction of Wenxia Formula Extract Inhibited MMP2 and MMP9 Expression: Histological and Biochemical Evidence

**FIGURE 4:**
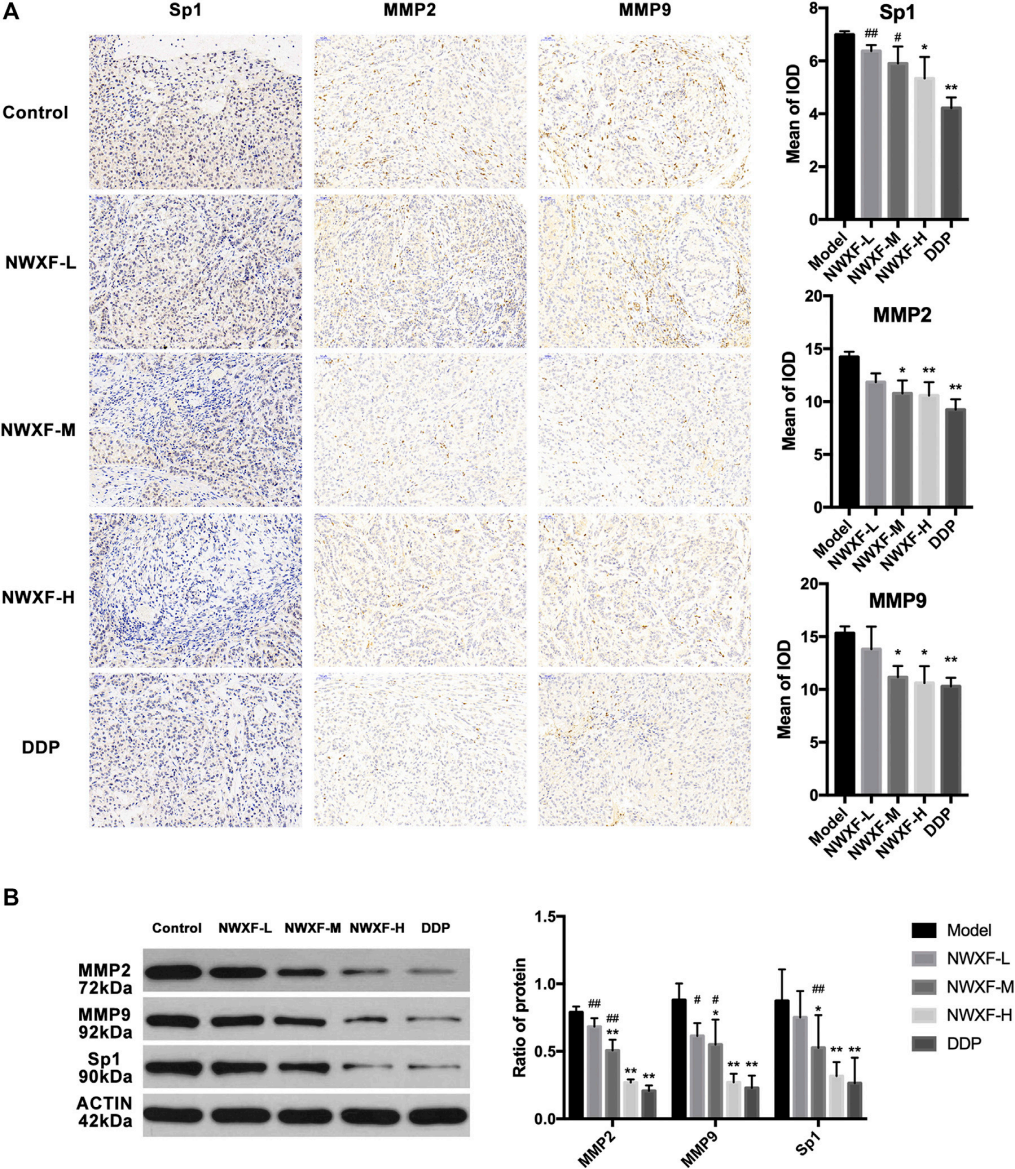
NWXF inhibits expression of Sp1, MMP2, and MMP9 in transplanted tumor. **(A)** Immunohistochemical detection of Sp1, MMP2, and MMP9 protein levels in orthotopic site (image magnification: ×200). The mean of iod were analyzed by Image-Pro Plus 6.0 software. **(B)** Western blot analysis of Sp1, MMP2, and MMP9 expression in xenograft. ^∗^ *p* < 0.05, ^∗∗^ *p* < 0.01 vs. the control group. ^#^ *p* < 0.05, ^##^ *p* < 0.01 vs. the DDP group.

To further confirm our results, immunohistochemical detection and western blot assay were performed to investigate whether NWXF could inhibit migration and invasion of A549 cells *in vivo*. The results showed that NWXF can down-regulate the expression of Sp1, MMP2, and MMP9 in tumor tissues (Figure 4).

### Chemical Profiling of N-Butanol Fraction of Wenxia Formula Extract

HPLC/MS analysis was employed to characterize the chemical composition of NWXF. A total of 201 compounds were putatively identified by comparing database mzCloud, of which 117 compounds had a mzCloud Best Match score greater than 80. Different kinds of ingredients are included in these compounds, such as polysaccharides, saponins, anthraquinone, organic acids and so on. Among them, ginsenoside and ferulic acid showed obvious anti-cancer effect. Mass spectrometry analysis was performed on certain compounds with mzCloud Best Match of over 80. The results are shown in Table 1.

**TABLE 1:** Characterization of some chemical constituents in NWXF by HPLC/MS Analysis.

Name	Formula	Molecular weight	RT (min)	mzCloud best match
DL-Tryptophan	C_11_H_12_N_2_O_2_	204.08959	6.396	99.4
Adenosine	C_10_H_13_N_5_O_4_	267.09622	5.033	98.5
Erucamide	C_22_H_43_NO	320.30654	25.6	95.9
Bis(2-ethylhexyl) phthalate	C_24_H_38_O_4_	390.2755	25.066	95.8
Catechin	C_15_H_14_O_6_	290.07857	6.646	95.7
Adenine	C_5_H_5_N_5_	118.02801	2.239	94.5
Dibutyl phthalate	C_16_H_22_O_4_	278.1513	17.962	94.2
Ferulic acid	C_10_H_10_O_4_	194.05769	7.735	93.1
1,6-Bis-O-(3,4,5-trihydroxybenzoyl) hexopyranose	C_20_H_20_O_14_	501.11129	6.872	92.7
Kaempferol	C_15_H_10_O_6_	286.0472	9.961	92.3
4-Coumaric acid	C_9_H_8_O_3_	164.04721	7.43	91.7
Nicotinic acid	C_6_H_5_NO_2_	123.03229	2.26	91.5
Emodin	C_15_H_10_O_5_	270.05252	10.909	89.7
Aloe-emodin	C_15_H_10_O_5_	270.05315	7.729	88.6
Ginsenoside Rg3	C_42_H_72_O_13_	766.4849	14.334	88.1
Linoleic acid	C_18_H_32_O_2_	280.23986	21.075	85.8
Aconitine	C_34_H_47_NO_11_	645.31433	11.902	85.7
Salsolinol	C_10_H_13_NO_2_	179.09432	3.435	85.5
Cytarabine	C_9_H_13_N_3_O_5_	243.08539	2.154	85.5
Ginsenoside Rb1	C_54_H_92_O_23_	1,130.585	11.173	84.5
Kaempferol-7-O-glucoside	C_21_H_20_O_11_	448.10029	9.368	84.5
Formononetin	C_16_H_12_O_4_	268.07305	10.503	82.3
Sedanolide	C_12_H_18_O_2_	194.13054	12.095	81.8
Citric acid	C_6_H_8_O_7_	192.02603	1.611	80.8
Betulin	C_30_H_50_O_2_	442.3801	14.324	80.2

## Discussion

Based on the theory of traditional Chinese Medicine, effective extraction and compatibility of traditional Chinese Medicine have attracted more and more attention from the medical profession (Bordoloi et al., 2016; Zhang et al., 2018; Mahmoudian-Sani and Asadi-Samani, 2020; Tian et al., 2020). The WXF is a traditional Chinese medicine formula consisted of multiple components, which focuses on dispelling cold, supplementing yangqi, activating blood circulation and clearing blood stasis. It was observed that the active ingredients of the WXF components such as aconitine (Qi et al., 2018), ginsenoside Rh2 (Li et al., 2018; Li X. et al., 2020), Rg3 (Sun et al., 2017; Xiao et al., 2019), emodin (Zhang et al., 2019a) and Angelica polysaccharide (Zhang et al., 2019a) have anti-tumor activities. Previous studies have shown that WXF has specific anti-tumor properties (Zhang et al., 2019b), and that extracts of WXF retain these anti-tumor effects.

In this study, NWXF was separated by silica gel column chromatography in order to explore the properties of the active anti-tumor compounds in WXF. The chemical profile of NWXF was characterized by UPLC/MS analysis and a total of 201 compounds were identified. Furthermore, we aimed to evaluate the tumor-suppressing effect of NWXF *in vitro* and *in vivo*. *In vitro* experiments, NWXF inhibits tumor cell proliferation in a dose-dependent manner. Considering that excessive concentrations of NWXF inhibit the migration and invasion of NSCLC cells mostly due to the cell damage by NWXF. We set up the 100 μg/ml, 200 μg/ml, and 400 μg/ml NWXF group in the migration and invasion assay. In Figure 1A, NWXF has showed little cytotoxicity in NSCLC cells at 100 μg/ml for 24 h. In Figure 1B, NWXF could significantly inhibit the migration and invasion of NSCLC cells at 100 μg/ml for 24 h. In view of this, We hold that NWXF could inhibit migration and invasion of NSCLC cells. *In vivo* experiment, DDP was used as a positive control. Because of its strong and wide curative effect, it was recommended as the first choice for lung cancer. It has been reported that DDP treatment suppressed the MMP2 and MMP9 protein expressions significantly in human adenocarcinoma cell line A549 (Liang et al., 2013). We found that the weight and volume of xenograft in NWXF-treated group were significantly lower than those in the control group, indicating that NWXF could inhibit A549 growth *in vivo*. These were consistent with our previous study, which showed that NWXF could significantly reduce the tumor volume and alleviate the tumor weight. In addition, we found that NWXF extract had no significant effect on the spleen and body weight of mice, while the DDP group had significant spleen and body weight loss, indicating that NWXF was safer than DDP.

Transcription factor Sp1 is the founding member of the Sp family that perform a functional role in the regulation of so-called housekeeping genes (Beishline and Azizkhan-Clifford, 2015). Sp1-dependent transcription is regulated throughout cellular development, proliferation, differentiation and tumorigenesis (Safe et al., 2018). It is well documented that Sp1 is over-expressed in many cancers, including lung (Hu et al., 2019), breast (Dong et al., 2018), gastric (Xu et al., 2018) and colorectal (Liu et al., 2018) cancers. Sp1 levels correlate with invasive potential and metastasis, with high levels of Sp1 being related to poor prognoses (Chen et al., 2019). In patient samples, Sp1 levels are associated with the survival of cancer patients (Liu et al., 2016). Therefore, studies have addressed the possibility of targeting Sp1 to treat cancer cells (Sankpal et al., 2011; Bearz et al., 2019).

Multiple reports have shown that Sp1 was a key regulator in the transcription of MMPs and MMP-like proteins (Guan et al., 2012; Vizcaíno et al., 2015). Sp1 is probably recruited to the MMP2 promoter through interaction with the SWitch/Sucrose NonFermentable complex, which is essential for the activation of the MMP2 promoter, subsequently increasing the mRNA and protein expression of MMP2 (Beishline and Azizkhan-Clifford, 2015). Over-expression of both MMP2 and MMP9 proteases requires the transcriptional activity of Sp1. In addition, MMPs is greatly recognized as a booster of tumor growth in tumorigenesis and participate in degrading mechanical barrier such as extracellular matrix and basement membrane to facilitate cell movement (Li K. et al., 2020). It is well documented that the proteolytic activities of MMPs are involved in the metastasis process, including enabling cell adhesion, migration, and invasion. Among them, MMP2 and MMP9 have long been suspected of performing essential roles in human malignancies and are associated with reduced survival and poor prognosis in NSCLC (Jayakumar et al., 2018).

In the present study, we demonstrated that NWXF efficiently suppressed the migration and invasion of A549 and H460 cells confirmed by transwell migration and invasion assay. Interestingly, at effective doses up to 100 μg/ml, cell proliferation was little affected, but rather increased in a dose-dependent manner, indicating that NWXF is a safe anti-tumor component. In addition, levels of MMP2 in 100 μg/ml NWXF-treated A549 and H460 cells were respectively lower to those of NWXF-untreated cells, indicating that NWXF has inhibitory effect on the migration and invasion of NSCLC cells.

In studies examining the mechanism of anti-migration action, we observed that NWXF treatment had no influence on Sp1 binding to the MMP9 promoter. In contrast, NWXF treatment inhibited the binding activity of Sp1 to the MMP2 promoter, resulting in decreased MMP2 expression, which is crucial for cancer migration and invasion. The overall results provide evidence that NWXF inhibits A549 and H460 cells migration and invasion by down-regulating Sp1-mediated MMP2 expression.

In conclusion, we found that NWXF prevents NSCLC cells against migration by suppression of Sp1 activation and decreased the accumulation of MMP2 and MMP9 expression. Further studies to investigate the mechanism of NWXF on Sp1 are needed. More importantly, animal models of metastatic need to be carried out to observe the anti-metastasis effect of NWXF. The findings of this study suggest that NWXF has the potential to be helpful for the treatment of NSCLC.

## Data Availability Statement

The raw data supporting the conclusions of this article will be made available by the authors, without undue reservation.

## Ethics Statement

The animal study was reviewed and approved by Animal Welfare and Ethics Committee of Shandong University of Traditional Chinese Medicine for Nationalities.

## Author Contributions

XJ conceived the study, participated in the design and coordination of the whole study, and helped in critically revising the draft for important intellectual content. QB and MRW carried out the majority of the experiments and drafted the manuscript. FZ and MW participated in the data collection and helped in the animal experiments. XY, JR, and DW participated in several experiments and performed the statistical analysis. All authors read and approved the final manuscript.

## Funding

This study was supported by the National Key R&D Program of China (Nos. 2019YFC1708700, 2019YFC1708702) and the National Natural Science Foundation of China (Nos. 81774198, 81703839). The Open Project of Shandong Co-Innovation Center of Classic TCM Formula also supported the work (No. 2018KFZ03).

## Conflict of Interest

The authors declare that the research was conducted in the absence of any commercial or financial relationships that could be construed as a potential conflict of interest.
