# Supplementary material for: N-Butanol Fraction of Wenxia Formula Extract Inhibits the Growth and Invasion of Non-Small Cell Lung Cancer by Down-Regulating Sp1-Mediated MMP2 Expression
Source: Front Pharmacol. 2020 Nov 30;11:594744. doi: 10.3389/fphar.2020.594744 (PMC7734278; doi:10.3389/fphar.2020.594744)
Supplement: Supplementary file 1 [file table1.doc]

Supplementary Table 1.Detailed information of the crude drugs composed in WCF

| No | Composition of Drugs | Dose | Origin of medicinal materials | Herbal medicine processing plant | Voucher Specimens Reserve |
| --- | --- | --- | --- | --- | --- |
| WCF4-A | Aconitum carmichaeli Debeaux | 3000g | Taiping Town,  Jiangyou City,  Sichuan Province  W:104.75°  N:31.78° | Anhui Province Xiehe pharmaceutical Yinpian Co., Ltd.  No. 2008, Yaodu Road, Qiaocheng District, Bozhou City, Anhui Province  W:115.78°  N:33.85° | Affiliated Hospital of Shandong University of Traditional Chinese Medicine |
| WCF4-B | Rheum palmatum L. | 3000g | Shajin Town,  Li County,  Gansu Province  W:105.15°  N:34.22° | Anhui Province Xiehe pharmaceutical Yinpian Co., Ltd.  No. 2008, Yaodu Road, Qiaocheng District, Bozhou City, Anhui Province  W:115.78°  N:33.85° | Affiliated Hospital of Shandong University of Traditional Chinese Medicine |
| WCF4-C | Panax ginseng C.A.Mey | 2250g | Beigang Town,  Fusong County,  Jilin Province  W:127.28°  N:42.33° | Anhui Province Xiehe pharmaceutical Yinpian Co., Ltd.  No. 2008, Yaodu Road, Qiaocheng District, Bozhou City, Anhui Province  W:115.78°  N:33.85° | Affiliated Hospital of Shandong University of Traditional Chinese Medicine |
| WCF4-D | Angelica sinensis (Oliv.) Diels | 1500g | Meichuan Town,  Min county，  Gansu Province  W:104.04°  N:34.41° | Anhui Province Xiehe pharmaceutical Yinpian Co., Ltd.  No. 2008, Yaodu Road, Qiaocheng District, Bozhou City, Anhui Province  W:115.78°  N:33.85° | Affiliated Hospital of Shandong University of Traditional Chinese Medicine |
